# Oral health status and barriers to oral healthcare among children with cerebral palsy attending a health care center in Kampala, Uganda

**DOI:** 10.1186/s12903-022-02677-2

**Published:** 2022-12-30

**Authors:** S. M. Kachwinya, A. M. Kemoli, R. Owino, I. Okullo, J. Bermudez, A. L. Seminario

**Affiliations:** 1 School of Dentistry, Makerere University, Kampala, Uganda; 2 School of Dental Sciences, University of Nairobi, Nairobi, Kenya; 3 Northwest Dental Residency, Advanced Education in General Dentistry Program, Yakima, WA USA; 4 School of Dentistry, School of Public Health, University of Washington, Seattle, USA; 5 School of Dentistry, Universidad Peruana Cayetano Heredia, Lima, Peru

**Keywords:** Cerebral palsy, Oral hygiene, Oral diseases, Comprehensive Rehabilitation Services Uganda (CoRSU)

## Abstract

**Background:**

Cerebral palsy (CP) is a non-progressive neuromuscular condition diagnosed in childhood. CP as a form of disability, does not cause any specific oral disease. However, some oral conditions are more commonly associated with people with CP compared to the general population. The overarching aim of the current study was to determine the oral hygiene status, gingival status, and the prevalence of dental caries in children with CP attending a leading hospital institution for children with disabilities in Kampala, Uganda. Additionally, we determined the barriers faced by children with CP in accessing oral healthcare.

**Methods:**

This cross-sectional study was carried out at the Comprehensive Rehabilitation Services Uganda hospital in Kampala, Uganda. Our study population consisted of a convenient sample of 90 children diagnosed with CP aged 3–17 years and their caregivers. A validated and interviewer administered structured questionnaire was used to collect socio-demographic data of the participants. A modified World Health Organization oral health assessment form for those aged 3–17 years was used to gather data on oral health status (plaque score, gingival bleeding and dental caries.) The data was subjected to statistical tests with critical value set up at 5%.

**Results:**

Only 32.2% of the children evaluated had adequate oral hygiene, while 44.4% of the children experienced gingival bleeding. The prevalence of dental caries for both deciduous and permanent dentition was 63.3%, with DMFT values of 3.8 ± 4.5. The most common barrier reported by the caregivers was the challenge in modality of transportation availability from the children’s homes to the health facilities (34.4%).

**Conclusions:**

Children with CP in the study population have a significant prevalence of oral diseases and face several barriers to oral healthcare. Results from this study aim to provide relevant support to advocate for a nationwide change in policy to improve access to dental care to decrease the burden of oral diseases in children with special healthcare needs.

## Background

Cerebral palsy (CP) is a non-progressive neuromuscular condition diagnosed in childhood, and characterized by difficulties in mobility, coordination, and posture [1]. Children with CP will typically present with hypotonicity, spasticity, and abnormal coordination, with various levels of severity [2]. The April 2006 Definition and Classification of CP Report [2] classifies CP into subtypes, with all having atypical pattern of movement and poor posture in common. The spastic CP sub-type is the most common at 50–60%, followed by dyskinetic CP at 15%, and the ataxic is the least common sub-type at 7–15% of all CP cases.

CP is the most common physical disability, and is believed to be caused by atypical development of the brain or by trauma to the developing brain that results in the inability to control muscles [3]. The global prevalence of CP is 1.8–2.3 cases in 1000 live births [4] while the prevalence in Africa has been found to be at 2–2.5 cases in 1000 live births. The most common cause of CP in Africa is birth asphyxia, brain infections, and kernicterous [5]. In Uganda, a low income country in East Africa with a population of 42.7 million, children make up 60% of the population. A study in Uganda found the prevalence of CP was 2.7 per 1000 live births. After adjusting for attrition this prevalence increased to 2.9 per 1000 live births [6]. Therefore, children in Uganda have a two-fold risk of developing CP compared to children in high income countries.

CP is a physical disability and does not cause any specific oral disease. However, some oral conditions are more commonly associated with children with CP compared to the general population. This is because the poor manual dexterity associated with most children with CP contributes greatly to their inability for most of the children to manage their own or receive requisite oral hygiene measures like brushing and flossing. They, therefore, end up having poor oral health [7]. At the top of the list of the oral health disorders associated with CP are dental caries and gingivitis/periodontal disease (PD), both conditions resulting from poor oral hygiene as the primary risk factor [8, 9]. The general prevalence of dental caries in Ugandan children has ranged from 17 to 65% [10–13], and that of gingivitis ranging from 72 to 75% [11, 14], and this situation could be worse for children with CP. Most children with CP seek healthcare services from the public hospitals and clinics. The public healthcare system in Uganda is decentralized and provides general and oral health services at national and district levels. However, these health services are inadequate and inequitable, with the vulnerable and the rural communities receiving diminished services [15]. Children with CP thus face several barriers to oral healthcare, some of which are determined by geographic location of the children, the type of CP, and the availability of oral health care services [16]. These barriers can lead to accumulated untreated caries [17], which in turn affects the quality of life of the child. Identifying these barriers to oral healthcare access can provide a framework for intervention measures for this vulnerable community [18].

No research has been conducted on the oral health experience of children with CP in Uganda. The overarching aim of the current study was to determine the oral hygiene status, gingival status, and the prevalence of dental caries in children with CP attending a leading hospital institution for children with disabilities in Kampala, capital of Uganda. Additionally, we determined the barriers faced by children with CP in accessing oral healthcare. The study hypothesized that oral diseases would be present at least in half of the children with CP. The results of this study will provide baseline information to the policy makers on oral health challenges faced by children with CP that could help in the development of a framework for intervention.

## Methods

Ethical approval to study human subjects was obtained from the KNH-UoN Ethics and Research Committee, Kenya (P605/10/2017) and the MUKCHS Institution Review Board and Ethics Committee, Uganda (MAKSHSREC-REF:2018-021). This research was conducted in accordance with the ethical guidelines provided in the Declaration of Helsinki [19].

### Study design and population

This cross-sectional study was carried out in 2018 at the Comprehensive Rehabilitation Services Uganda (CoRSU) hospital in Kampala, Uganda. Children with CP visiting the hospital are attended to by a pediatrician during their weekly CP and neurology clinics, and benefit from the health education, pediatric review, physiotherapy, and surgery (contracture release) services offered by the center. At the time of the study, the total number of children with cerebral palsy and seeking care at CoRSU was 120. Eligibility for inclusion in this study included: (1) not presenting with any illnesses unrelated to CP (e.g. Malaria, diarrhoeal diseases and upper respiratory tract infections), (2) cooperative behavior for oral examinations, and (3) parents/guardians providing informed consent as well as assent (where possible) from the child. Our study population consisted of a convenient sample of 90 children (75% of population of interest) diagnosed with CP aged 3–17 years and their caregivers, and attending the clinics at CoRSU hospital during the study period of 15th May 2018 to 15th June 2018.

### Intervention

Prior to the commencement of the study, the principal investigator (PI) was calibrated by a senior oral health clinician (AMK) on how to determine plaque score (PS), gingival status (GS), and dental caries of ten children. Inter-examiner reliability for plaque score and gingival bleeding score were Kappa 0.89 and 0.77, respectively). The PI then trained 2 research assistants (RA) on their roles in the data collection process. One RA was trained and pretested on how to administer the questionnaire to the caregivers, while the second RA was trained and pretested on how to record data obtained during the clinical examination by the PI.

### Data collection tools

*Questionnaire*: A validated and interviewer-administered structured questionnaire adapted from the one used by Ober-Oluoch et al. [20], was used to collect socio-demographic data of the participants. The estimated monthly income of the caregiver was documented based on the Uganda National Household Survey 2016–2017 household earnings [21], data on the distances from their home to the nearest oral healthcare facility, and challenges faced with this journey were also documented. The distance to the nearest facility for oral health care services for the child was assessed in terms of minutes taken by bus or car to reach the facility or when walking.

### Oral examinations

A modified World Health Organization (WHO) oral health assessment form for those aged 3–17 years was used to gather data on oral health status [22]. Oral health assessments of the participants were conducted at CoRSU center under field conditions (natural light augmented by a headlamp) using the WHO guidelines [22, 23]. The examination was done with the child either sitting on the caregiver’s lap, who was seated on an ordinary chair, or where possible with the child sitting unaided in their own wheel chair or on an ordinary chair. Sterile instruments (a mouth mirror, a ball ended probe) and sundry materials (rubber gloves, paper hand towels, and gauze), were used during the oral examination.

Initially, a visual inspection of the gingival tissue and the hard tissues was done to score for presence of dental plaque (8 surfaces were scored and the sum recorded per tooth). The Silness-Loe plaque index was used and scored as 0–0.9 (absence of plaque), 1–1.9 (plaque along the free gingival margin), 2–2.9 (moderate accumulation of plaque up to one third of the tooth surface) and 3 (plaque covering more than one third of the tooth surface). The tip of a ball ended probe (periodontal probe WHO) was carefully inserted between the gingiva and the tooth, and walked around the surface of the tooth following the anatomical contour to determine the presence or absence of bleeding [22]. The teeth were then cleaned using sterile gauze and visual and tactile examination of each tooth was done to detect for the presence of dental caries in the form of decayed, missing and filled teeth (dmft/DMFT). Any signs of dental trauma and erosion observed on individual teeth were also documented. Persistent drooling was assessed by observing the prescence of excessive saliva pouring out of the participants mouth and stained clothing. Bruxism was assessed based on history of tooth grinding sounds made by the participants and observation of attrition of the occlusal surfaces of the teeth. All the forms used in the research data collection were reviewed by the principal investigator at the end of each day for their accuracy and completeness. The information was then entered into the database.

### Data analysis

The data collected were pre-coded, entered in Epi Data version 3.02, cleaned and then exported to the Statistical Package for Social Sciences (SPSS Inc., Illinois, Chicago, USA, Version 23.0 for Windows) for analysis. Descriptive statistics was conducted to summarize demographic variables. T-test, ANOVA, and chi-square tests were conducted to identify factors associated with oral health outcomes (plaque score, gingival bleeding, and dental caries). Critical value was set up at 5%.

## Results

### Socio-demographics

Our study population consisted of 90 children with CP aged 3–17 years (mean 7.1 ± 3.9 years); median 5y [IQR = 6]). Half of the participants were female (50.0%) while the majority of the children were first-borns (35.6%), 16.3% were only children, 5.0% had 1 sibling 21.3% had 2 or 3 siblings each, 18.8% had 4 siblings, 15.0% had 5 siblings and 2.2% had 6 siblings. The majority of children (85.5%) were born within health facilities, as reported by the caregivers. Additionally, the majority of caregivers (46.1%) were aged between 21 and 30 years and more than a third of them (35.6%) had attained a college-level education. More than half of the caregivers 57.3% were married, 15.7% were widowed, and most caregivers’ monthly income was below USD13.5 (44.8%) (Table 1).

**Table 1:** The social demographic characteristics of the study participants and their caregivers

Variable	Mean ± SD	Median (range)
Age (years)	7.1 ± 3.9	5 (3–17)
Birth weight (Kg)	3 ± 3.0	3 (1–4.8)
Number of siblings	3.3 ± 1.3	3 (1–6)

	Frequency N (%)
Sex	
Female	45 (50)
Male	45 (50)
Birth facility	
Home	3 (3.3)
Health facility	77 (85.5)
Don’t know	10 (11.1)
Caregivers’ age category	
20 years or younger	1 (11.1)
21–30	41 (46.1)
31–40	30 (33.7)
41–50	13 (14.6)
51 or older	4 (4.5)
Caregivers’ marital status	
Single	24 (27)
Married	51 (57.3)
Divorced/Separated/Widowed	14 (15.7)
Caregivers’ level of education	
Primary	27 (30)
Secondary	29 (32.2)
College	32 (35.6)
Don’t know	2 (2.2)
Caregivers’ monthly income	
< $13.50	44 (44.8)
$13.50–$27	11 (12.2)
$27–$54	8 (8.8)
> $54)	25 (27.7)

### Oral health status of the participants

**Fig. 1:**
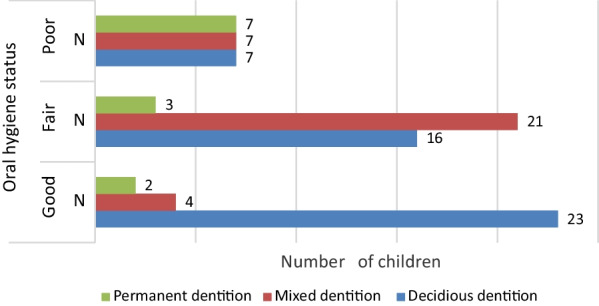
Oral hygiene status of the children in the study population

The oral health characteristics of the participants is shown in Table 2 and Fig. 1. A moderate overall mean plaque score was observed among participants [1.9 ± 0.74)], with 44.4% of them having gingival bleeding. This varied significantly in relation to the type of dentition (*p* < 0.05). Gingival bleeding was present in 77.0%, 70.0%, and 16.0% of the children in permanent dentition, mixed dentition, and primary dentition respectively. The plaque score was found to increase with the age of the children. We found the prevalence of dental erosion (14.4%) and dental trauma (12.2%) to be low among the study participants while drooling and tooth wear due to bruxism was found to be high at 70.0% and 52.0% respectively (Table 2).

**Table 2:** Oral health status of the participants

Variable	Mean ± SD
Plaque score (N = 90)	1.9 ± 0.7
DMFT	3.8 ± 4.4

Characteristic	Frequency (N)/%
Gingival bleeding	
Present	40 (44.4)
Absent	50 (55.6)
Dental erosion	
Absent	77 (85.6)
Present	13 (14.4)
Dental trauma	
Absent	76 (84.4)
Present	11 (12.2)
Other damage	3 (3.3)
Dental caries	
Yes	57 (63.3)
No	33 (36.7)
Dentition status	
Primary dentition	36 (40.0)
Mixed dentition	38 (42.2)
Permanent dentition	16 (17.8)
Other oral conditions	
Drooling	63 (70.0)
Bruxism	47 (52.2)

The overall prevalence of dental caries for both deciduous and permanent dentition was 63.3%, with the DMFT values of 3.8 ± 4.5. Specifically, 40.0% of participants in primary dentition had dental caries in average (mean dmft of 2.8 ± 4.3 while participants in mixed and permanent dentition had 42.2% and 17.8% affected, respectively (Table 3). When analyzing child and caregiver characteristics related with presence of dental caries, only the child’s age was significantly associated with dental decay in primary dentition (*p* = 0.0045). The younger children aged < 6 years had the highest dmft value of 3.8 ± 4.9, while the children aged 6–12 years had dmft 1.3 ± 2.00. In permanent dentition, mean DMFT values were significantly different among children aged < 6, 6–12, and more than 12 at [0.0 ± 0, 1.5 ± 3.3, 3.9 ± 4.7, *p* ≤ 0.001]. The caregivers’ age was also significantly associated with the prescence of dental decay in the permanent dentition with DMFT [0.0 ± 0.0, 0.3 ± 0.95, 1.0 ± 1.8, 3.5 ± 4.8, 2.2 ± 2.6 at *p* = 0.008] at ages < 20, 21–30, 31–40, 41–50 and > 51 respectively. Participants with caregivers aged 41–50 had the highest DMFT (Table 3). The socio-economic information, such as level of education and monthly income of the caregivers is also provided in Table 3.

**Table 3:** Caries experience in primary and permanent teeth by the socio-demographic characteristics among the participants

Variable	N (%)	dmft		DMFT	
		Mean ± SD	*p* value	Mean ± SD	*p* value
Sex					
Male	45 (50.0)	3.1 ± 4.0	0.5269	0.7 ± 0.24	0.123
Female	45 (50.0)	2.5 ± 4.6		1.5 ± 0.45	
Child’s age category					
< 6 years	57 (63.3)	3.8 ± 4.9	0.0045	0.28 ± 1.2	< 0.001
6–12 years	24 (26.7)	1.3 ± 2.0		1.6 ± 1.8	
> 12 years	9 (10.0)	0.0 ± 0.0		4.5 ± 5.4	
Birth order					
First birth position	32 (35.6)	2.7 ± 4.0	0.678	0.8 ± 2.9	0.359
Other birth positions	48 (53.3)	3.1 ± 4.8		1.4 ± 2.3	
Don’t know	10 (11.1)	1.2 ± 2.1		0.5 ± 1.1	
Caregivers’ age category					
20 years or younger	1 (1.1)	0.0 ± 0.0	0.970	0.0 ± 0.0	0.0008
21–30	41 (46.1)	2.9 ± 4.5		0.3 ± 0.95	
31–40	30 (33.7)	2.9 ± 4.2		1.0 ± 1.8	
41–50	13 (14.6)	2.5 ± 4.4		3.5 ± 4.8	
51 or older	4 (4.5)	3.0 ± 3.8		2.2 ± 2.6	
Caregivers’ Level of education					
Primary	27 (30.0)	3.4 ± 5.1	0.6231	0.9 ± 2.3	0.737
Secondary	29 (32.2)	2.9 ± 4.4		0.9 ± 1.8	
College	32 (35.6)	2.3 ± 3.6		1.3 ± 3.2	
Don’t know	2 (2.2)	1.0 ± 1.4		1.0 ± 1.4	
Caregivers’ Marital status					
Single/unmarried	38 (42.7)	3.1 ± 4.6	0.6482	1.2 ± 3.1	0.675
Married	51 (57.3)	2.6 ± 4.1		1.0 ± 1.8	
Caregivers’ Monthly income					
< $13.50	44 (48.9)	3.2 ± 4.5	0.421	0.8 ± 1.7	0.7204
$13.50–$27	11 (12.2)	3.3 ± 4.6		1.4 ± 3.1	
$27–$54	7 (7.9)	1.3 ± 1.3		1.8 ± 2.5	
> $54	25 (27.9)	5.7 ± 9.0		1.2 ± 3.4	
Uncomfortable responding	3 (3.3)	6.0 ± 8.3		0.7 ± 1.2	

### Access to oral healthcare and barriers to access

Caregivers believed seeking dental care was only for emergency treatment, such as swelling (22.2%), cracked teeth with pain (5.5%), and for mobile teeth (8.9%). The average time from home to the nearest oral health facility was 34 ± 25. The minimum time reported was 1 min when using motorized transportation, while the minimun time taken to walk to the facility was 45 min. The maximum time for both walking and using motorized transportation was 120 min. The average cost of vehicular (bicycle, boda boda, public van taxis, buses and private special hire cars) fare was $1.13 ± $1.1. The lowest cost was $0.13, while the highest was $8.10 (Table 4).

**Table 4:** Barriers to accessing oral health care among the study participants

Barrier	Mean ± sd (mins)/ Median ± range (mins)
Time taken to reach the facility by bus/car	34 ± 25
Time taken to reach facility on foot	45 ± 120
Transport cost to facility	$1.1 ± 1.1/$0.13 ± 8.1

	Frequency (N/%)
Rate of the cost in terms of affordability(n = 22)	
Very high	3 (9.5)
High	3 (14.3)
Fair	12 (57.1)
Low	4 (19.0)
Child related problems (the primary health condition and child unwilling to cooperate)	20 (22.2)
Transportation and dstance to oral healthcare facility	31 (34.4)
High financial costs of dental treatment	27 (30.0)
Service provider issues (difficulty finding a dentist willing to provide care)	5 (5.7)
Fear of pain at the dental visit	7 (7.7)

## Discussion

The goal of this study was to determine the oral health status of children living with CP in Kampala, Uganda and the barriers they face in accessing oral health care.. We hypothesized that oral diseases would be present in at least half of our study population. Our findings showed that only 32.2% of the participants had adequate oral hygiene, with 44.4% presenting with gingival bleeding. The prevalence of dental caries for both, deciduous and permanent dentition was 63.3%, with DMFT values of 3.8 ± 4.5. The most common barrier that caregivers reported was the challenge in transportation availability to health facilities (34.4%). Our results confirmed our hypothesis that oral diseases were high in half of the study population of children with CP seeking care at the Comprehensive Rehabilitation Services Uganda Hospital in Kampala, Uganda.

The 90 children with CP, who participated in. the study, were aged 3–17 years (mean = 7.1 years, median = . 5 years) and the majority were first-borns and had 2 or 3 siblings. According to the World Bank, Uganda has a high fertility rate of 5.4, which varies depending on area of residence and region, with the rural women having an average rate of 5.9 children compared to 4 among urban ones [24]. While about one third of caregivers (35.6%) had attained a college degree, almost half of them earned less than $13.5 per month and about a quarter were earning above $54 monthly. In our study population, a majority of the children’s primary caregivers were predominantly mothers who may be unable to remain in formal or informal employment as a result of both the child’s condition and large fertility rates, among other reasons. The 2016/2017 Uganda National Household Survey 2, reported that only 19.3% of adult female workers got theirwages from employment while the majority (39.6%) were subsistence farmers [21]. This socio-demographic profile exhibited in our study population varied from the one conducted in India, and which reported 89.7% of the mothers having been housewives, in spite of 61% having attained a tertiary level education [25].

We found that the majority of the children (67.8%) had fair to poor oral hygiene. The results are slightly lower than those reported by Ober-Oluoch et al. [26] who had 73.68% children with fair to poor oral hygiene in a Kenyan pediatric population. This could be attributed to the fact that the study by Ober et al. was carried out in a dental camp setting while the present study was carried out in a hospital setting, whereby the caregivers of our study were being exposed to healthcare workers who gave them general hygiene information, including oral health education. With a similar setting to the current study, the Egyptian study reported that 61.3% of the children with CP had fair to poor oral hygiene [27], which was consistent with the findings in our study. We also found that plaque accumulation increased with age; children aged 14 years and above had the highest level of plaque accumulation. This was consistent with existing evidence that teenagers tend to have higher plaque scores, resulting from the influence of the behavioral changes that occur during puberty, a time they are less inclined to observe proper oral hygiene practices [28]. Plaque accumulation was also found to be significantly associated with the number of siblings (*p* = 0.013). Possiblly, the more siblings in the family the more challenges the cargeivers had to address in relation to the oral health needs of the child with CP. this situation makes the caregiver be unable to efficiently ensure adequate plaque control for the child with CP.

Bleeding was present in 44.4% of the children. Evidence exists that the prevalence of gingivitis will be up to three times higher in children with CP than in normal children [29]. While our findings of gingivitis were lower than the Kenyan study by Ober-Oluoch et al. [26], who found the prevalence of moderate and severe gingivitis to be 55.8% and and 10.4% respectively, they were however similar to those of Egyptian study reported at 43.5% in children aged 3–12 years [27]. Unlike the Kenyan study, both, our study and the Egyptian study were carried out in a hospital setting where the caregivers had some exposure to general hygiene education.

The prevalence of dental caries in the study population was high at 63.3%, witha dmft of 3.8 ± 4.5). This was comparable to the prevalence of 66.3% dmft of 5.41in a Kenyan study of children with CP [26]. In both studies, the decayed component was the highest and there were the unfilled teeth. The lower dmftI h in the current study may be attributed to the fact that the participants in our study had mild to moderate forms of CP, which could have allowed them to eat healthy, noncariogenic food and possibly brush their teeth. Specifically, the main source of food in the Ugandan’s low socioeconomic group is home-grown subsistence, non-processed, and has low cariogenicity such as steamed banana, boiled maize flour and stewed beans. The more severe form of CP, the higher the likelihood of dysphagia which limits the child’s diet to sugary semi solid carbohydrate porridges [30]. In the primary dentition, children younger than 6 years of age had significantly higher dmft (*p* = 0.0045).

Aance to dental health services was low with only 32.6% of the children having ever visited a dentist. Caregivers believed seeking dental care was only for emergency treatment, such as swelling (22.2%), cracked teeth with pain (5.5%), and for mobile teeth (8.9%). This low utilization of oral health services is a common practice in low income regions and is not merely restricted to children with CP [31]. The most common barrier reported by caregivers in the current study were difficulty in transportation of the children from their homes to the health facilities. Most of these caregivers used public systems and had to carry their children (child with CP and siblings) aboard mini buses and buses often needing connecting connecting busses to reach the hospital. Unfortunately, in children with disabilities such as CP the caregivers have to handle children above the age of 3 years who may weigh up to more than 15 to 20kgs. Our findings were similar to the Kenyan study by Ober-Oluoch et al. in 2011 which reported that 55% of caregivers of children with CP had difficulty accessing dental treatment due to the high cost of fare. The low income of the majority of the caregivers presents a major barrier considering that the highest cost of transportation to an oral health facility was USD8.1 in the present study. This is more than half of their monthly income to access oral healthcare. Our findings are consistent with a 2022 Brazillian study that looked at aged 3–18 year-old children with CP. The study found that children with more severe forms of CP had least favourable socioeconomic backgrounds and the most common accessibility barrier was transportation challenges [32]. These barriers can be overcome by primarily raising awareness of oral health conditions and spreading preventive messages to non-oral health workers who are more accessible to these children, such as their medical doctors, nurses, physiotherapists, and nutritionists.

This study has several limitations. Firstly, the low sample size. While the hospital at which these study subjects were recruited is the largest of its kind in the region, it only averages about 15 children with CP a week. Yet, we were successful at recruiting all individuals who sought care during the time period of our study. Secondly, some of the children could not participate in the study due to the severity of their disabilities. As such the children with severe forms of CP with severe behavioral limitations or very ill with any other disease unrelated to CP could not be examined and were under-represented in this study. Finally, this was a cross-sectional study and there was no data on the diet of the participants. However, the primary diet of the demographics in the catchment area of the hospital consists of organic carbohydrates, legumes, and leafy green vegetables prepared by steaming and stewing [21].

## Conclusion

The present study shows that children with CP in Uganda have a high prevalence of oral diseases and face several barriers to oral healthcare, most of which are determined by the geographic location of the children and accessibility to oral health services. These findings call for public policies that target caregivers of children with CP to increase access to dental care by facilitating transportation services that include young siblings who are under the attention of the caregiver. These barriers can lead to poor oral hygiene, impaired gingival health, accumulated untreated caries, and impact their overall health. The high levels of oral disease among children with CP call for increasing awareness and strategies on interventions that target oral health care at hospital settings. Results from this study aim at providing relevant support to advocate for a nationwide change in policy to improve access to dental care to decrease the burden of oral diseases in children with special healthcare needs.

## Data Availability

The data that provide the basis for the presented results of this study is available by contact to the corresponding author, but restrictions apply to the availability of these data and to a certain time period, as the data were used under license for the current study, and so are not publicly available.

## Abbreviations

ANOVA: Analysis of Variance
CORSU: Comprehensive and rehabilitation services Uganda
CP: Cerebral palsy
DMFT/dmft: Decayed, Missing, Filled teeth
MUK: Makerere University Kampala
PI: Principle Investigator
PhD: Doctor of Philosophy
SD: Standard deviation;
SCPE: Surveillance of Cerebral Palsy in Europe
WHO: World Health Organisation
